# Continuous Audio‐Visual Sensor Monitoring Is More Effective Than Human Observers for Detecting Moor Macaques

**DOI:** 10.1002/ajp.70121

**Published:** 2026-01-20

**Authors:** Víctor Beltrán Francés, Anja Hutschenreiter, Hjalmar S. Kühl, Federica Amici, Risma Illa Maulany, Putu Oka Ngakan, Bonaventura Majolo, Denise Spaan

**Affiliations:** ^1^ Instituto de Neuroetología Universidad Veracruzana Veracruz México; ^2^ Instituto de Investigaciones en Ecosistemas y Sustentabilidad Universidad Nacional Autónoma de México Morelia México; ^3^ Senckenberg Museum for Natural History Görlitz, Senckenberg Member of the Leibniz Association Görlitz Germany; ^4^ International Institute Zittau Technische Universität Dresden Zittau Germany; ^5^ Fakultät Biologie Technische Universität Dresden Germany; ^6^ Life Sciences, Institute for Biology, Human Biology and Primate Cognition Leipzig University Leipzig Germany; ^7^ Department of Comparative Cultural Psychology Max Planck Institute for Evolutionary Anthropology Leipzig Germany; ^8^ Forestry Department Hasanuddin University Makassar Indonesia; ^9^ School of Psychology, Sport Science and Wellbeing University of Lincoln Lincoln UK

**Keywords:** camera traps, detectability, passive acoustic monitoring, point transects, population surveys

## Abstract

The number of species threatened with extinction is continuously increasing, underscoring the need for reliable population estimates to develop effective conservation plans. The ability to confirm a species' presence during surveys (i.e., detectability) is central for population estimates. While audio‐visual sensors, like camera traps and passive acoustic monitoring (PAM), have emerged as valuable tools for monitoring primates, few studies have systematically compared their detectability, particularly in dense forests with limited visibility and for elusive species. Here, we compared 40‐days continuous monitoring with audio‐visual sensor (camera traps, *N* = 19; PAM, *N* = 7) versus human‐based point transects with three survey visits (*N* = 20) on wild moor macaques (*Macaca maura*) in two different habitats: forest (*N* = 10) and open areas (*N* = 10). Using occupancy models to compare the detection probability (*p*), we found that camera traps (*p* = 0.63 ± 0.04) and PAM (*p* = 0.79 ± 0.08) outperformed point transects (*p* = 0.33 ± 0.07), regardless of habitat type. After equalizing survey time between methods, we found that detections were greater on point transects in surveys shorter than 1 day, but camera traps and PAM equalized their performance with two survey days (*p*‐value < 0.05). Notably, combining both audio‐visual sensors yielded the highest detectability (*p* = 0.87 ± 0.05). These results highlight the effectiveness of audio‐visual sensors and support multi‐method approaches for monitoring primates in tropical forests. Overall, this research contributes to designing more effective monitoring protocols for primate species, which are essential for planning conservation strategies.

## Summary

- Camera traps and acoustic monitoring over 40 days detected moor macaques more effectively than 3‐visit human surveys, with both sensor methods showing similar detectability.
- Under 1‐day surveys, human point transects detected moor macaques better than audio‐visual sensors. Beyond 2 days, sensors exponentially increased detectability, surpassing transects after 15 days.
- Combining camera traps and PAM maximized moor macaque detectability. Results support using both methods for primate surveys in tropical forests, confirming audio‐visual sensors as a key conservation tool.

## Graphical abstract

Camera traps and PAM outperformed human transects in detecting moor macaques: sensors overcame point transect detectability after 2 days survey. Combining both audio‐visual methods achieved highest detectability, supporting audio‐visual sensors as essential tools for primate monitoring in tropical forests.

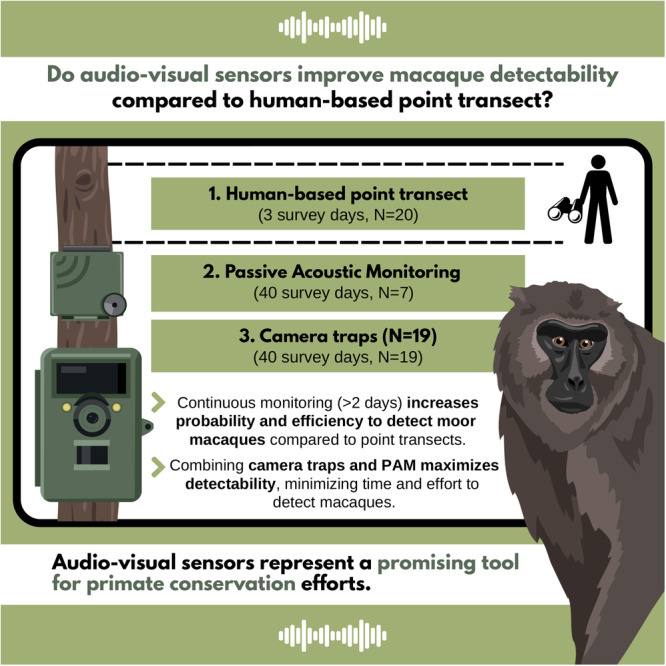

## Abstract (id)

Jumlah spesies yang terancam punah terus mengalami peningkatan, sehingga semakin menegaskan pentingnya estimasi populasi yang ada dalam penyusunan rencana konservasi yang efektif. Salah satu aspek krusial dalam estimasi populasi adalah kemampuan untuk mengkonfirmasi keberadaan suatu spesies selama survei (kemampuan deteksi). Meskipun sensor audio‐visual, seperti kamera jebak dan pemantauan akustik pasif (Passive Acoustic Monitoring‐PAM), telah berkembang sebagai alat yang penting dalam pemantauan primata, namun penelitian secara sistematis yang membandingkan efektivitas deteksi kedua metode ini masih sangat terbatas, khususnya pada hutan yang cukup rapat dengan visibilitas rendah dan pada spesies yang sulit ditemukan. Dalam penelitian ini, kami membandingkan pemantauan berkelanjutan selama 40 hari menggunakan sensor audio‐visual (kamera jebak, *N* = 19; PAM, *N* = 7) dengan metode transek titik berbasis manusia yang dilakukan melalui tiga kali kunjungan survei (*N* = 20) pada populasi monyet dare (Macaca maura) di dua tipe habitat: hutan (*N* = 10) dan area terbuka (*N* = 10). Dengan menggunakan model okupansi untuk membandingkan probabilitas deteksi (p), hasil menunjukkkan bahwa kamera jebak (*p* = 0,63 ± 0,04) dan PAM (*p* = 0,79 ± 0,08) memiliki kinerja lebih baik dibandingkan transek titik (*p* = 0,33 ± 0,07), tanpa dipengaruhi oleh tipe habitat. Setelah waktu survei disetarakan antar metode, ndeteksi lebih tinggi pada transek titik untuk survei yang berlangsung kurang dari satu hari. Namun, kamera jebak dan PAM menunjukkan kinerja setara setelah dua hari survei (*p* value < 0,05). Menariknya, kombinasi kedua sensor audio‐visual menghasilkan tingkat keterdeteksian tertinggi (*p* = 0,87 ± 0,05). Temuan ini menegaskan efektivitas sensor audio‐visual dan mendukung pendekatan multi‐metode dalam pemantauan primata di hutan tropis. Secara keseluruhan, penelitian ini berkontribusi pada desain protokol pemantauan yang lebih efektif bagi spesies primata, yang sangat penting dalam perencanaan strategi konservasi.

**Abbreviations**
ARU: Autonomous recording unit
PAM: Passive acoustic monitoring

## 1 Introduction

The high level of anthropogenic activities in natural ecosystems is leading to the sixth mass extinction (Ceballos et al. 2020). Defaunation is especially critical in tropical forests, which are home to at least two thirds of all terrestrial species (Barlow et al. 2018). These habitats host more than 300 primate species threatened with extinction due to deforestation largely caused by agricultural and forestry industries (Carvalho et al. 2019; Estrada et al. 2017). In the current situation, conservation agencies need tools to assess the status and trends of primate populations in the short and long term (Lindenmayer et al. 2022) to implement efficient conservation measures (Stephenson et al. 2022). Monitoring programs can provide estimates of the occupancy, density, or abundance of a population, but such population estimates are highly influenced by the detectability of the monitored species (i.e., the probability of detecting the presence of a species in the sampled area; Guillera‐Arroita 2017). When detectability is modeled during statistical analysis, higher detectability can lead to estimates with smaller confidence intervals and therefore more reliable population estimates (i.e., accurate and precise; Royle et al. 2005). Monitoring protocols with higher detectability may thus contribute to a more informed understanding of species‐habitat relationships and aid in the design of effective conservation plans that are crucial to address the current extinction crisis (Wang et al. 2024).

Different factors can affect the detectability of a species, including its anatomical and behavioral traits (e.g., body size, activity and locomotion patterns; Sólymos et al. 2018; Wessling and Surbeck 2023), its ecological characteristics (e.g., habitat, weather; Ramellini et al. 2024), the methods used to monitor the species (e.g., survey method; Tucker et al. 2021), and the interactions between all these factors (Anderson et al. 2015; Einoder et al. 2018). Species detectability, for instance, may vary when using the same survey method in different habitats (Missa et al. 2009). In tropical forests, visibility may be low due to dense vegetation, rainfall or steep slopes, leading to low visual species detectability compared to open, dry or flat areas (Willson et al. 2011). Moreover, the use of visual methods is advantageous to detect large, abundant or group living species (Marshall et al. 2008), while acoustic methods may aid the detection of more elusive species with frequent or loud acoustic activity (e.g., *Nomascus hainanus*; Dufourq et al. 2021). Failing to account for these differences in detectability by applying an appropriate survey method may severely bias population estimates, challenging our understanding of species‐habitat relationships (MacKenzie and Royle 2005). Such bias is especially relevant for threatened species living in habitats undergoing fast and constant anthropogenically‐induced modifications (Burns et al. 2019).

Traditionally, primates have been monitored using human‐based transects, in which an observer searches for visual and/or acoustic signals of the target species to determine their presence and/or to estimate their population density (Buckland et al. 2010; Hutschenreiter et al. 2021; Spaan et al. 2020). Both line and point transects are labor‐intensive methods and are usually challenging to deploy on a large scale in tropical forests, not only due to difficulties associated with topography and weather, but also because of the high economic costs and administrative complexities when working across multiple political jurisdictions during long field‐work periods (Plumptre et al. 2013, but see Bessone et al. 2022 for large scale surveys in lowland forests). Moreover, visibility plays a key role during transects, especially in dense tropical forests, where the maximum visual detection distance is relatively low (Buckland et al. 2010). As animals may flee quickly upon hearing or seeing humans, observers need to be skilled at rapidly detecting and identifying the target species, to avoid misidentification (which may increase both type I and type II errors; Buckland et al. 2010) and minimize biases in distance measurements.

The development of new technologies has provided conservation practitioners and the scientific community with alternative methods to detect primate species (Piel et al. 2021), including camera traps (e.g., Haysom et al. 2021; Hu et al. 2023) and passive acoustic monitoring (PAM; e.g., Kalan et al. 2015). Both camera traps and PAM can collect data continuously (i.e., 24 h/day) and autonomously (i.e., without the presence of humans; Piel et al. 2021), which reduces fieldwork effort and increases survey area, overcoming the limitations of traditional transects and facilitating the development of larger‐scale monitoring programs in ground‐dwelling primate species (Campos‐Cerqueira and Aide 2016; Wägele et al. 2022). As a visual detection method, camera trap detectability is influenced by similar factors as traditional direct observation methods like transects (Burton et al. 2015). Nevertheless, camera traps are motion‐activated by a passive infrared monitoring trigger (PIR; Kays et al. 2021), which in turn adds new sources of variability to the detection probability (Rovero and Zimmermann 2016). For example, species‐specific parameters, including body size (Tobler et al. 2008), locomotion (i.e., terrestrial or arboreal; Fang et al. 2018), and daily ranging patterns (Rowcliffe et al. 2016) may affect detectability in camera trap studies, in addition to other factors such as camera trap model, orientation and height from the ground (Palencia et al. 2022).

Factors that affect PAM detectability include those that increase environmental noise or reduce target sound propagation (Marten and Marler 1977; Zwerts et al. 2022). Background noise from roads or human presence on acoustic recordings can complicate species identification (Heinicke et al. 2015), and physical barriers, such as dense vegetation or rock walls may lead to poor sound propagation (Marten and Marler 1977). Audio recorders should thus be placed in areas with as few physical obstacles as possible (Sugai et al. 2019). Climatic factors may also affect detectability, as warm temperatures and low humidity favor sound propagation, whereas heavy rain and strong wind increase background noise (Kalan et al. 2015).

In forest areas, camera traps and PAM can detect primates with higher probability than transects (Hutschenreiter et al. 2022; Moore et al. 2020), because of the longer survey times (i.e., duration of time in which the devices are active; Cappelle et al. 2021; Zambolli et al. 2022). Although the spatial effort (i.e., total area surveyed) of transects is usually larger than that of a single camera trap or an audio recorder, the high mobility of primates may favor camera trap and PAM detectability, if the survey time is sufficient to capture individuals as they move through their home range (Després‐Einspenner et al. 2017). Conversely, detectability in open areas may be similar when using transects or camera traps, as the transect survey area increases exponentially with visibility (Buckland et al. 2010). PAM, however, may detect primates in open areas better than either method, as the maximum detection distance of acoustic signals increases due to sound transmission compared to forest areas (MacLaren et al. 2018). Given that detectability varies with survey methodology and habitat type, it is important to test which methodology best detects the species of interest, to standardize methods and avoid biases in population estimates resulting from the methodology being used (Ruiz‐Gutiérrez and Zipkin 2011).

Despite the increasing use of audio‐visual sensors to monitor primate populations, comparisons of detectability across different audio‐visual methods and habitats are lacking, especially in dense forests with reduced visibility and for more elusive primate species (Piel et al. 2021). While there are studies comparing primate detectability between transects and camera traps (Cappelle et al. 2019; Li et al. 2012; Moore et al. 2019), transects and PAM (Hutschenreiter et al. 2022; Kalan et al. 2015), and camera traps and PAM (Enari et al. 2019; Crunchant et al. 2020), there is, to our knowledge, no research comparing the detectability of the three methodologies in more than one habitat for primate species.

## 2 Description

The main objective of our study was to compare the detectability of three different survey methods (camera traps, PAM, and human‐based point transects) to monitor the endemic and threatened moor macaque (*Macaca maura*) in two different habitat types (forest vs. open areas). Restricted to the highly deforested region of Sulawesi Selatan in Indonesia (Riley et al. 2020; Supriatna et al. 2020), moor macaques occupy fragmented landscapes comprising forest patches and open areas, including agricultural and urban zones. While traditional monitoring has relied on transect surveys (Beltrán Francés et al. 2022; Supriatna et al. 1992), the species' semi‐terrestrial behavior and vocalizations suggest that audio‐visual sensor methods could enhance detection.

Camera traps may be effective given the macaques' frequent ground movement (Zak and Riley 2017), while PAM could capitalize on male loud calls, a high‐frequency (4–6 kHz), multi‐syllabic vocalization produced during fights, group movement, and at dawn and dusk (Okamoto and Matsumura 1998; Thierry et al. 2000). Moreover, both audio‐visual sensor methods could enhance macaque detectability by enabling continuous 24‐h data collection, which is particularly valuable as macaques can be active during nocturnal and crepuscular periods (Zak and Riley 2017), when human‐based transect surveys are hindered by low‐light conditions. In forested environments, both audio‐visual sensor methods might outperform point transects, where limited visibility and observer presence may reduce macaque detectability in human‐based point transects. Conversely, in open habitats, enhanced visibility and improved sound propagation might increase the effectiveness of point transects to levels comparable with camera traps while maintaining PAM's superior detectability.

To compare these methods, we surveyed the detectability of moor macaques in forest and open areas using standardized protocols based on fieldwork feasibility and species' behavior and ecology: camera traps (visual detection) and PAM (audible detection) collected data 24 h/day over 40 consecutive days (with one survey defined as 5 days of sampling), whereas point transects (visual and audible detection) involved three 200‐min surveys between 6:00 and 18:00 and within the same period. As survey effort differed between each design, we further assessed whether equalizing survey time influenced detectability. Our findings provide practical insights into the strengths and limitations of each method, facilitating the development of targeted monitoring strategies that optimize fieldwork efficiency and data reliability for moor macaques and ecologically similar primate species.

## 3 Example

### 3.1 Ethics Statement

Our study did not involve any direct contact with moor macaques, as our methods only included observations and audio recordings. During point transects and while setting up the devices, we always kept a minimum distance of 10 m from any moor macaque encountered, following the American Society of Primatologists Code of Best Practices for Field Primatology. The Indonesian “Badan Riset dan Inovasi Nasional” (BRIN) approved our study and the protocol we used to collect field data (research permit number: 209/SIP/IV/FR/10/2022). This research adhered to the American Society of Primatologists Principles for the Ethical Treatment of Nonhuman Primates.

### 3.2 Study Population

**Figure 1:**
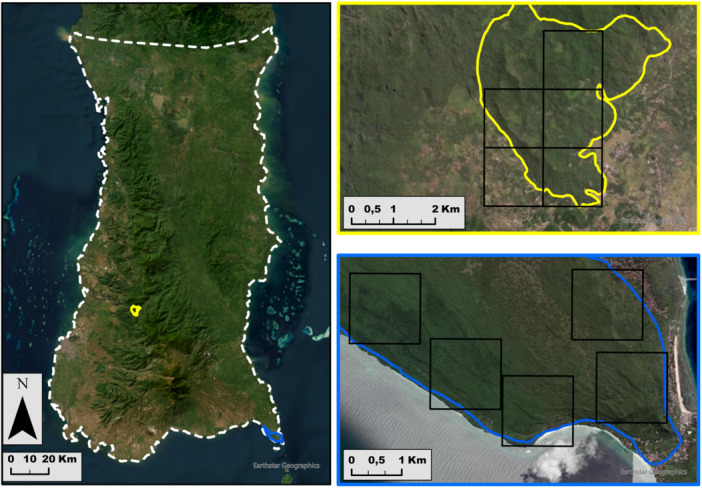
Map of the moor macaque geographic distribution (left; white dashed line; Riley et al. 2020) and the sampling locations (2 km^2^; black squares) in the two areas where the study took place: Hutan Pendidikan (yellow) and Wildlife Reserve Taman Hutan Rakyat Bontobahari (blue).

The geographic distribution of moor macaques is restricted to the province of Sulawesi Selatan in the southwest of Sulawesi Island, Indonesia. The species ranges from the Tempe depression in the north (3°47'55.29“S, 120° 3'32.41“E) to the southern tip of the province in Bontobahari (5°33'1.33“S, 120°24'45.78“E; Figure 1). For this study, we collected data in two different areas: the Hutan Pendidikan and the Wildlife Reserve Taman Hutan Rakyat (TAHURA) Bontobahari. In both areas, several groups of moor macaques have been studied since 2017 (Hernández Tienda et al. 2022; Rahman et al. 2024). The Hutan Pendidikan in Bengo (5°00'S, 119°46'E, Limapoccoe, Cenrana, Maros Regency) is a 1300‐ha protected area managed by the Forestry Faculty of the Hasanuddin Universitas (Makassar, Sulawesi Selatan, Indonesia). This area ranges between 400 and 800 m above sea level and presents a rainy season of around 5 months (3000 mm/year; November‐March). The second area corresponds to the TAHURA Bontobahari (5°36'S, 120°26'E, Darubiah, Bulukumba Regency), a 3475‐ha protected area composed of savannah forest, ranging between 0 and 100 m above sea level and with a 3‐month long rainy season (2500 mm/year; December‐February). Both regions included forest patches surrounded by open areas such as agricultural fields and human settlements, permitting the collection of data in both forested and open areas.

### 3.3 Experiment 1

#### 3.3.1 Data Collection and Processing

From the 21st of November 2022 to the 12th of January 2023, we collected data on the presence of macaques using point transects, camera traps, and PAM. In each study area (i.e., Hutan Pendidikan and TAHURA Bontobahari), we selected 5 sampling locations to survey the detectability of moor macaques (Figure 1). We defined a sampling location as a 2 km^2^ square plot containing both forested and open habitat types, where the presence of moor macaques had been previously confirmed (Beltrán Francés et al. 2022). Inside each sampling location, we defined a survey site as the precise location where we deployed one survey method to collect data on the presence of moor macaques. Therefore, each sampling location contained between one (i.e., a single survey method deployed in one habitat type) and six survey sites (i.e., all three survey methods deployed in both forest and open areas). As previous research obtained a maximum visual detection distance of 64 m for moor macaques during line transect surveys in forested areas (Beltrán Francés et al. 2022), we selected 100 m as a conservative minimum radius distance from the center of the sampling point to differentiate between forest (i.e., the entire 100 m radius of the point transect was composed of forest) or open areas (i.e., absence of forest cover within a 100 m radius of the point transect center).

##### 3.3.1.1 Point Transects

**Figure 2:**
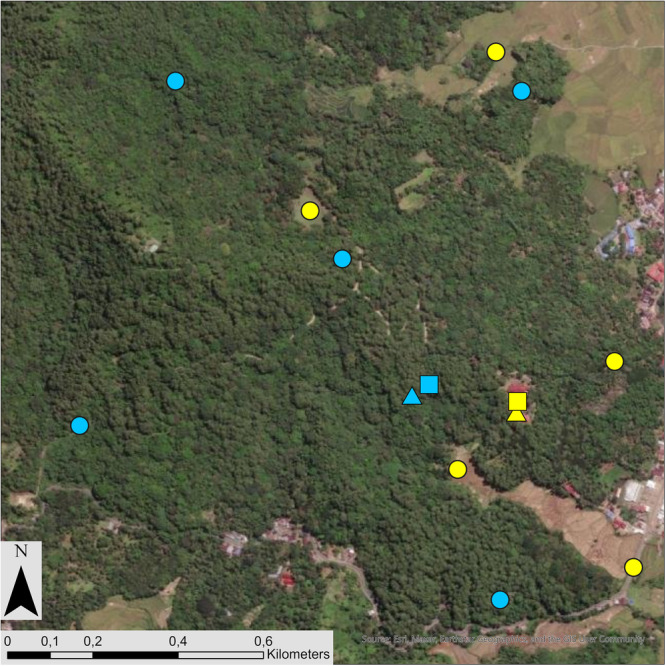
Distribution and location of survey sites for point transects (dots; i.e., five sampling points surveyed for each point transect), camera traps (squares; *n* = 1 unit), and autonomous recording units (ARUs; triangles; *n* = 1 unit) at one sampling location in Hutan Pendidikan. Blue symbols represent locations in forested areas, and yellow symbols represent locations in open areas.

To test moor macaque detection probability during human‐based point transects, we conducted one point transect per habitat type (forest and open) within each sampling location. We defined one point transect (i.e., one survey site) as a set of five randomly distributed sampling points. To randomly place the sampling points of each point transect, we first selected forest and open area polygons within each sampling location. Secondly, we used the “Create Random Points” tool in ArcGIS Pro (3.3.2, ESRI) to distribute five sampling points in each habitat type, with a minimum distance of 500 m between points to ensure data independence (Buckland et al. 2010; Figure 2). When the forest or open area within one sampling location was not big enough to distribute five sampling points, we located the highest possible number of points while maintaining the minimum distance between sampling points.

We surveyed a total of 20 point transects (i.e., 10 in Hutan Pendidikan and 10 in TAHURA Bontobahari; 91 sampling points) three times each during a 40‐day period (MacKenzie et al. 2017). We separated two consecutive visits by at least 5 days (Buckland et al. 2010). To cover the complete range of activity periods of the species, we surveyed point transects between 06:00 and 18:00 (Beltrán Francés et al. 2022). On each transect, we waited 20 min to detect the presence of macaques at each sampling point before walking to the next point (Hutschenreiter et al. 2021). We walked the distance between sampling points at 2 km/h (to reduce disturbance that might affect the presence of the species at a sampling point). We marked all sampling points with a GPS device (Garmin Etrex 10x) so that we could visit the exact same sampling point during subsequent visits. We considered “macaques detected” at a point transect if at least one moor macaque was visually or audibly detected in at least one of the five sampling points. Otherwise, we classified the point transect as “macaque not detected”. We did not code species as present in the point transect if the macaque was detected while walking the distance between two sampling points.

##### 3.3.1.2 Camera Traps

To test the detection probability of moor macaques using camera traps, we located two camera traps at every sampling location (i.e., 10 camera traps in the area of Hutan Pendidikan and 9 camera traps in the area of TAHURA Bontobahari; 3 Bushnell Trophy Cam HD, 10 Bushnell Core 24 MP No Glow, and 6 WingScapes WCW‐00120). At each sampling location, we located one camera trap in the forest area and one camera trap in the open area (e.g., crop field) closest to the center point of the sampling location (Figure 2). We placed no camera trap in the open area of one sampling location at TAHURA Bontobahari due to safety concerns. We set each camera trap at 30 cm height from the ground with high sensitivity, to ensure maximum detectability (Beltrán Francés, unpublished data). We programmed camera traps to take three pictures each time they were triggered and with a delay of 5 s between two consecutive triggers (Cavada et al. 2019). To avoid activation and save battery life and memory, we cleared all herbaceous plants taller than 30 cm within a 2 m range of each camera trap's field of view (Rovero et al. 2010). The camera traps collected data on at least 40 consecutive days at each sampling location (Rovero et al. 2014). We defined one survey occasion as five consecutive days of camera trap data (Johnson et al. 2020). Therefore, each camera trap had eight survey occasions per survey site (Rovero et al. 2014). We considered “macaque detected” on a survey occasion if at least one individual was detected in a photo (Johnson et al. 2020), otherwise, we coded it as “macaques not detected”.

##### 3.3.1.3 Passive Acoustic Monitoring

To test the detection probability of moor macaques using PAM, we deployed a total of seven autonomous recording units (ARUs; AudioMoth) across five sampling locations in both study areas. Specifically, we located four ARUs in forest areas and two in open areas in Hutan Pendidikan and one in a forest area in TAHURA Bontobahari. We located ARUs in the forest and open areas closest to the center point of the sampling location (Figure 2). Due to the limited number of ARUs available at the beginning of the data collection period (three ARUs), we did not distribute the ARUs homogeneously between sampling locations, nor for the same amounts of time. We deployed three ARUs during 40 consecutive days (from 1st December 2022 until 10th January 2023) and four ARUs during 15 consecutive days (from 28th December 2022 until 12th January 2023). To avoid ground interference and increase the probability of loud call detections, we placed each ARU at 2 m above the ground (Batist et al. 2024). Due to problems with the lifespan of the batteries used, not all ARUs collected data for the entire time deployed in the field. The three ARUs deployed during 40 consecutive days collected data for a mean of 32 days (range: 24–40 days), and the four ARUs deployed during 15 consecutive days collected data for a mean of 7 days (5–11 days). We programmed ARUs to record for 50 s every minute, for 24 h a day (sampling rate = 16 kHz).

To identify and detect the loud call vocalizations of the species from the audio recordings collected in the field, we used the software Ocenaudio (v3.11.22) to visually inspect the spectrograms and listen to the 1591.41 h of audio recorded. As for camera traps, we defined one survey occasion as five consecutive days of ARU data (Johnson et al. 2020) and each ARU had eight survey occasions per survey site. We coded one survey occasion as “macaque detected” if at least one loud call vocalization was detected in one audio recording, otherwise, it was coded as “macaque not detected”.

##### 3.3.1.4 Combined Survey Methods

To evaluate detection probabilities across survey method combinations (i.e., point transects+camera traps, point transects+PAM, camera traps+PAM and point transects+camera traps+PAM), we compiled integrated detection histories for all sampling locations surveyed with multiple methods. For each survey occasion, we classified the survey as “macaque detected” when at least one method detected a macaque, otherwise recording “macaque not detected”.

#### 3.3.2 Data Analysis

##### 3.3.2.1 Detection Probability Modelling

We used single‐season occupancy models to analyze the effects of habitat type (forest vs. open area) and survey methodology (point transect, camera trap, PAM) on the detection probability of moor macaques (MacKenzie et al. 2017). Single‐season occupancy models estimate the probability of occupancy (ψ) and the probability of detection (*p*) of a single species using the detection history of one species in a sampled area during one season (MacKenzie et al. 2017). A season is defined as the period of time in which the species' population remains closed (i.e., no births, deaths, emigration or immigration of individuals are assumed to happen; MacKenzie et al. 2017).

For each model, we analyzed data from 86 different survey sites with a total of 20 point transects, 19 camera traps, 7 PAM, 19 point transects+camera trap, 7 point transect+PAM, 7 camera trap+PAM, and 7 point transects+camera trap+PAM. We included habitat type (i.e., forest and open area) and survey method (i.e., point transect, camera trap, PAM, and the different combinations of survey methods) as covariates of detection probability. As we detected moor macaques with at least one method in each of the 10 sampling locations surveyed, and we were only interested in the factors that affect macaque detectability, we considered ψ = 1 for the analysis and only modelled *p* (MacKenzie et al. 2017). In total, we ran 4 different models: the null model (i.e., without covariates), one model including only survey method as detection covariate, one including only habitat type, and one including the interaction of survey method and habitat type. We used the package unmarked (Fiske and Chandler 2011) in R (RStudio, 2020; version 2022.02.0) with the function “occu” to estimate moor macaque detection probability. We ranked the models according to the Akaike's Information Criterion corrected for small sample sizes (AICc) and considered the most parsimonious models those with ΔAICc < 7 (Burnham et al. 2011). Moreover, we ascertained that the null‐model ΔAICc was > 7 to rule out that the null model explained the data better than any of the model combinations with covariates (Burnham et al. 2011). To determine the likelihood that the observed data were described by the selected model(s), we used the “parboot” function to run the Chi‐square goodness‐of‐fit test for the selected model(s) (MacKenzie and Bailey 2004). Finally, we back‐transformed the detection probability estimates and their standard errors from the most parsimonious models, using the “predict” function in the unmarked package to visualize the results with the package ggplot2 in R. Following Kery (2002), we estimated the minimum number of survey occasions needed (N_min_) to detect moor macaques with 95% confidence for each method following the formula:

$$N_{\min}=\;\log\left(0.05\right)/\;\log(1–p)$$

Where *p* is the detection probability estimated for each method from the most parsimonious model.

##### 3.3.2.2 Potential Survey Area

For each method, we estimated the potential survey area (i.e., potential area covered during one survey) as a function of the maximum detection distance for each specific method (Tucker et al. 2021). As point transects and PAM rely on acoustic cues and are omnidirectional (i.e., can detect acoustic signals in all directions; Joel et al. 2024), we defined the potential survey area for both methods as the area covered by a circumference with 130 m radius, which is the maximum distance at which the moor macaque loud call is audible (Beltrán Francés, unpublished data). As point transects consisted of five sampling points, we multiplied the area covered by one point by five to estimate the complete area covered during one point transect. For camera traps, a unidirectional method relying on visible cues (Joel et al. 2024), we defined the effective survey area as the area covered by a circular sector of 23 m radius and 35° angle (i.e., maximum detection distance and opening angle provided by the camera trap manufacturer; Moeller et al. 2022).

##### 3.3.2.3 Economic Costs

We estimated the total economic cost to survey a single location using each survey method. Daily personnel costs, based on prices and mean salary in Sulawesi Selatan for 2025, were estimated at 50 USD per person (5 USD for food, 15 USD for lodging, 20 USD for gasoline and vehicle renting to transport from basecamp to sampling location and 10 USD for the local field assistant salary). For audio‐visual sensors, we considered a half‐day salary (5 USD), as fieldwork only involved deploying and picking up devices and was therefore considerably shorter than for point transects. The total personnel cost was calculated by multiplying this daily rate by the number of field days required to survey a single location per method: 3 days for point transects (150 USD) and 2 days for audio‐visual sensor deployment (90 USD; 1 day to install the equipment and 1 day to remove it). For camera traps and PAM, we also included the cost of one device (100 USD for both methods) and the cost of Ni‐MH rechargeable batteries: six batteries for camera traps (10 USD) and three batteries for PAM (5 USD).

##### 3.3.2.4 Post‐Processing Time Effort

We estimated the post‐processing time required to convert raw field data into confirmed moor macaque detections for each method. For point transects, this involved transcribing field notes into a systematic digital format. We estimated this to take 1 h. For the audio‐visual sensor methods, we estimated the time needed to process the data generated over the standard 40‐day survey period. We calculated post‐processing times for both manual annotation and supervised automatic detection for both methods (Cowans et al. 2024). We estimated that manual annotation of camera trap data would require 8 h and supervised automatic processing would take 3 h. For PAM data, we estimated that manual annotation would take 40 h and supervised automatic processing would take 16 h.

##### 3.3.2.5 Experiment 2

###### 3.3.2.5.1 Survey Time Effect

To analyze the effect of survey time on macaque detectability (i.e., time period that the devices were active in the field), we calculated moor macaque detectability of camera traps and PAM across progressively increasing survey durations. We examined different windows of the survey time of camera traps and PAM in increments of 5 h, starting from 5 h up to 24 days per survey site. We selected a minimum survey window of 5 h to match the total time that we spent surveying one point transect (i.e., one point transect = 3 surveys x 1.67 h/survey = 5 h/point transect). As we surveyed three times each point transect, we limited the comparison to three randomly selected sampling occasions (1 sampling occasion = survey window/3) within the 40‐day period for camera traps and PAM to equalize survey time between methods. Consistent with point transect protocols, sampling occasions were also spaced at least 5 days apart. To estimate confidence intervals in detectability of both methods in each survey window, we repeated 1000 times the random selection of sampling occasions for each method and survey window. We coded the detection/not‐detection of the macaques at every survey occasion and used an ANOVA to analyze how survey time affected camera trap and PAM detection probability. For PAM, we used data from three sampling locations, as the remaining four collected data for less than 15 consecutive days, and therefore we could not perform the subsampling process for three survey occasions with at least 5 days between surveys.

### 3.4 Results

#### 3.4.1 Experiment 1

##### 3.4.1.1 Naive Detection

For the complete set of sampling locations surveyed (*n* = 10), we detected moor macaques in 96% of the forest survey sites (22/25), and in 76% of the open survey sites (16/21). For the eight survey sites where we did not detect moor macaques, we had surveyed seven of them with point transects and only one (in open area) with camera traps. Regarding survey occasions, during point transects we detected moor macaques in 30% (9/30) of the survey occasions in forest areas and 33% in open areas (10/30). For camera traps, we detected moor macaques in 68% (49/72) of the survey occasions in forest areas and 53% in open areas (34/64). For PAM, we detected moor macaques in 79% (15/19) of the survey occasions in forest areas and 80% (8/10) in open areas. While point transects mostly detected macaques in the early morning (06:00 to 09:00), PAM detected macaques around sunrise and sunset (05:00 to 07:00 and 17:00 to 19:00), when the frequency of loud calls usually peaks, and camera traps detected macaques from sunrise until early night (05:00 to 20:00; S1). Finally, we needed only one survey occasion to detect moor macaques using PAM, while we needed 1.3 survey occasions for camera traps (min = 1, max = 2) and 1.8 for point transects (min = 1, max = 3; Table 1).

**Table 1:** Number of point transects conducted and camera traps and autonomous recording units (PAM) employed in forest and open areas.

	Point transects	Camera traps	PAM
Forest (N)	10	10	5	
Open (N)	10	9	2	
Survey time (hours)^a^	1.67	120	120	
Survey area (ha)^b^	26.55	0.02	5.31	
Costs (USD)^c^	150	200	195	
Post‐processing time (hours)^d^	1	8 (3)	40 (16)	
Detection signal^e^	Audible/Visual	Visual	Audible	
Survey occasions (N)^f^	3	8	8	
Latency (mean; Min‐Max)^g^	1.8 (1–3)	1.3 (1–2)	1 (1–1)	
Detection probability (*p* ± SE)^h^	0.33 ± 0.03	0.65 ± 0.04	0.79 ± 0.08	
N_min_ (N)^i^	7.45	2.87	1.90	

^a^ Duration of one survey occasion.

^b^ Potential area surveyed during one survey.

^c^ Economic cost for 40 survey days (in USD calculated for October 2025).

^d^ Post‐processing time effort required to codify raw data into species detection per site (for camera traps and PAM automatic annotation within parenthesis).

^e^ Number of survey occasions for 40 days.

^f^ Signal modality (audible/visual) used to detect moor macaques during surveys.

^g^ Mean survey occasions needed until first detection of moor macaques.

^h^ Detection probability (*p*) and standard error (SE) estimated for the most parsimonious model.

^i^ Number of survey occasions needed to detect moor macaques with 95% probability.

Compared to the traditional point transects, camera traps detected macaques during the same survey occasion at the same sampling location in 66% (33/50) of the surveys and PAM in 56% (5/9), while camera traps and PAM detected macaques in 14% (7/50) and 44% (4/9) of the surveys that point transects did not. Finally, point transects detected moor macaques in 20% (10/50) of the surveys where camera traps did not, and on no occasion did point transects detect moor macaques that PAM did not.

##### 3.4.1.2 Detection Probability Modelling

**Figure 3:**
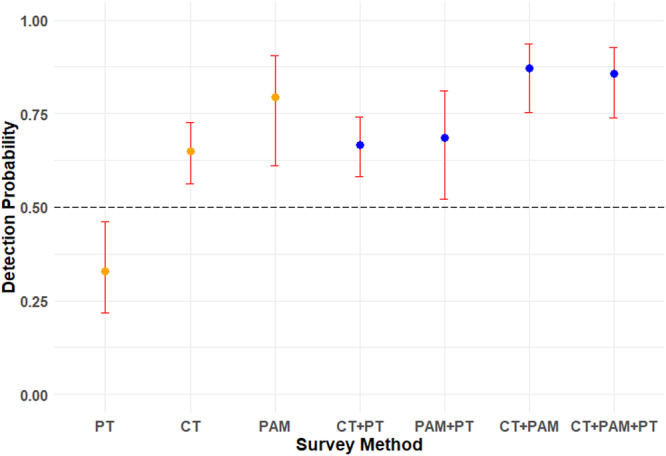
Estimated moor macaque detection probability from the most parsimonious detection model, with standard errors (red bars) for all the survey methods tested: single method per survey site (orange dots; point transects: PT, *N* = 20; camera traps: CT, *N* = 19; and passive acoustic monitoring: PAM, *N* = 7), at least two survey methods combined per survey site (dark blue dots; CT + PT, *N* = 19; PAM + PT, *N* = 7; CT + PAM, *N* = 7; CT + PAM + PT, *N* = 7). Dash line represents moor macaque detection probability of 50%.

The Chi‐square test (*p*‐value = 0.520) indicated a good‐fit for the most parsimonious model (i.e., the model including survey method as a *p* covariate; ΔAICc < 7; Table 2), suggesting that the selected model provided an adequate description of the data (Burnham et al. 2011) and that the survey method affected the detection probability of moor macaques in the wild. According to the ΔAICc comparison, there was no significant effect of habitat type on moor macaque detectability (Table 2), suggesting that detectability for the three methods is similar in forest and open areas. As only one model had ΔAICc < 7, we only used the parameters of this model to interpret the results (Burnham et al. 2011). Specifically, camera traps (*p* = 0.648 ± 0.042) and PAM (*p* = 0.793 ± 0.075) had significantly higher values of moor macaque detection probability than point transects (*p* = 0.329 ± 0.064), while camera trap and PAM did not show significant differences in moor macaque detection probability (Table 3; Figure 3). Although not statistically significant, the superior detectability of PAM should be interpreted with caution, as camera traps performed similarly at the seven survey sites where ARUs were deployed (S2). While the combination of camera traps and PAM (camera traps+PAM; *p* = 0.870 ± 0.046) detected moor macaques with higher probability than only camera traps (*p*‐value = 0.004), the combination did not significantly improve PAM detectability (*p*‐value = 0.359), but it contributed to shortening the detection probability confidence intervals (Figure 3). Point transects, conversely, did not improve single camera trap or PAM moor macaque detectability when combining methods (point transect+camera traps: *p*‐value = 0.755; point transects+PAM: *p*‐value = 0.322). According to detection probability estimates, point transects need at least twice as many survey occasions to detect moor macaques with 95% confidence as camera traps and PAM (Table 1), while the combination of camera traps and PAM needed only 5 days of data collection (N_min_ = 1.096).

**Table 2:** Model selection results for covariate effects of moor macaque detection probability in the two study areas (i.e., Hutan Pendidikan and TAHURA Bontobahari).

Model	NPar^a^	AICc^b^	ΔAICc^c^	*W*_*i*_ ^d^	Cumulative *W*_*i*_ ^e^
***p* (Survey method) ψ(.)**	**8**	**612.96**	**0**	**1**	**1**
*p* (Survey method * Habitat) ψ(.)	15	628.79	15.83	0	1
*p* (.) ψ (.)	2	642.57	29.61	0	1
*p* (Habitat) ψ (.)	3	643.44	30.48	0	1

*Note:* The model selected as the most parsimonious based on ΔAICc values is highlighted in bold.

^a^ Number of parameters.

^b^ Akaike information criterion corrected for small sample sizes.

^c^ Relative difference in AICc values compared to top‐ranked model.

^d^ AICc model weight.

^e^ Cumulative AICc model weight.

**Table 3:** Summary of conditional model averaged parameters based on the most parsimonious model.

Parameter	Estimate ± SE	*z* value	P (>|*z*|)
Intercept	−0.719 ± 0.288	−2.49	0.013*
*p* (CT)	1.330 ± 0.343	3.88	0.001*
*p* (PAM)	2.063 ± 0.542	3.81	0.001*
*p* (CT + PT)	1.412 ± 0.341	4.13	0.001*
*p* (PAM + PT)	1.493 ± 0.453	3.30	0.001*
*p* (CT + PAM)	2.624 ± 0.497	5.28	0.001*
*p* (PT + CT + PAM)	2.511 ± 0.479	5.25	0.001*

*Note:* Estimates of the β coefficient are reported for standardized covariates (scaled to mean = 0 and unit variance of 2). For the pairwise comparisons the reference level was point transects.

Abbreviations: CT, camera traps; PAM, passive acoustic monitoring; PT, point transects.

* Significant.

#### 3.4.2 Experiment 2

##### 3.4.2.1 Survey Time Effect

**Figure 4:**
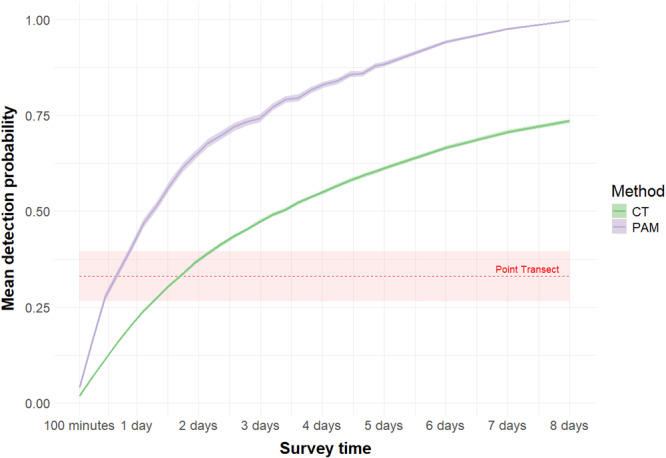
Mean moor macaque detection probability (line) and confidence interval (shadow) for camera traps (CT; green) and PAM (purple) with increasing survey time. In red, the mean detection probability (dashed line) and confidence interval (shadow) of point transects estimated with the occupancy model (i.e., survey time of one point transect equal to 5 h).

After subsampling the detection history of each method to different survey windows, we found that moor macaque detection probability of point transects (*p *= 0.331 ± 0.066) was higher than camera traps (*p *= 0.018 ± 0.001) and PAM (*p *= 0.042 ± 0.002) when survey time was equal between methods (5 h). However, both sensor‐based methods (camera traps: 1.5 days; PAM: 0.8 days) needed fewer than 2 days to equalize point transect detection probability, and fewer than 15 consecutive days (camera traps: 14.1 days; PAM: 5.1 days) to reach a detection probability higher than point transects in three survey days (*p*‐value < 0.05; Figure 4).

## 4 Comparison and Critique

To design effective monitoring programs for wildlife populations, it is necessary to understand how specific methods and habitats affect wildlife detectability. This is especially true under the current scenario, where the ongoing deforestation is dramatically affecting the distribution and population of threatened primate species in the tropics (Junker et al. 2020). Our results show how survey methods can affect primate detection probability. Using the presented survey design and irrespective of habitat type, both camera traps and PAM detected moor macaques more effectively than point transects, as they required fewer survey occasions to detect wild macaques. When survey time was limited (< 1 day), human‐based point transects detected moor macaques with higher probability than audio‐visual sensors. However, both audio‐visual methods outperformed point transects with extended survey durations (> 2 days). Although survey methods can detect differences across habitats, we found no significant effect of habitat type on detection probability for any of the three methods assessed. The similar detectability between camera traps and PAM, along with the high macaque detectability achieved when both methods are combined at a single survey site, suggests that integrating these audio‐visual techniques could enhance survey effort and primate detection rates, improving the monitoring of rare and endangered species.

Survey methodology can significantly affect species detectability (Joel et al. 2024; Li et al. 2012), potentially leading to inaccurate population estimates (MacKenzie et al. 2017). Our comparison of three feasible survey designs for moor macaques, 40‐day continuous camera trap and PAM deployments versus human‐based point transects with three survey visits, revealed that temporal sampling effort outweighed spatial coverage in determining detection success (Figure 3). While all methods assessed presence of macaques at fixed locations, continuous audio‐visual monitoring achieved higher detectability than point transects, despite covering fewer locations (1 vs. 5; Table 1), likely because moor macaques frequently use large portions of their home range during a week (i.e., 7 days; Hanya et al. 2020; Riley 2008). The better performance of audio‐visual sensors was particularly evident during crepuscular periods, when macaque activity peaks but low‐light conditions hinder human‐based surveys (S1), highlighting how automated methods can overcome temporal sampling limitations inherent to observer‐based approaches (Hutschenreiter et al. 2021). These findings suggest that an effective survey design for primates depends on both survey time and species' movement ecology. Extended monitoring (> 5 days) favors audio‐visual sensors by continuously monitoring presence, while brief surveys may benefit from point transects that concentrate effort in time (Figure 4). These methodological trade‐offs emphasize that researchers must carefully match survey design to both the behavioral ecology of the target species and the practical constraints of field conditions to maximize detection probability and data quality (Zwerts et al. 2021).

Contrary to our predictions, we found no effect of habitat type on moor macaque detectability. Although moor macaques are more frequently detected in forested areas (Beltrán Francés et al. 2022), and survey method detectability can vary between forest and open habitats (MacLaren et al. 2018), we found similar macaque detectability across both habitat types for all three methods. This lack of difference in detectability between habitats may be attributed to behavioral changes in moor macaques related to the habitat. In open areas such as agricultural fields or natural grasslands, the risk of encountering predators (e.g., humans, dogs) is higher, and macaques increase their vigilance, active foraging, and locomotion levels (Dhawale et al. 2020; Priston et al. 2012). This may lead macaques to show a more active behavior in open areas, which could enhance detectability even if they spend more time in forest areas. The lack of evidence for the predicted impact of habitat type may also depend on the limited number of PAM devices we located in open areas (Table 3). The unbalanced survey design resulted in large confidence intervals for PAM in open areas, which could explain the absence of an interaction effect of survey method and habitat type (MacKenzie et al. 2017). Interestingly, most of the survey sites where we did not detect moor macaques corresponded to point transects in open areas. As humans chase macaques away in both study sites, when they enter agricultural fields, these results might reflect the fact that, although audible range increases in open areas, macaques can detect humans from larger distances and flee before humans can detect them (Ikeda et al. 2022).

When high detectability is required for species monitoring, a multi‐method approach can significantly enhance detection rates and spatial coverage (Zamora‐Marín et al. 2021). Our results demonstrate that combining camera traps and PAM optimizes moor macaque detectability (Figure 3), enabling reliable species detection with reduced survey duration. The comparable detectability of both methods, moreover, would allow flexible deployment across different locations, increasing the number of available devices. These strategic approaches might enhance survey efficiency and increase spatial sampling, a critical factor for robust population monitoring and distribution analyses (Rovero and Zimmermann 2016). In addition, a multi‐method survey design may improve species detectability when the survey method is appropriately matched to local habitat conditions (Clare et al. 2017; Moll et al. 2023). On the one hand, PAM could improve the detectability of macaques in mountainous areas, where steep slopes can negatively affect detectability with camera traps (Shaw et al. 2021). On the other hand, the use of camera traps could improve detectability in areas with higher human disturbance, where soundscapes with high background noise may hinder species detection with PAM (Hoefer et al. 2023).

Although audio‐visual sensor methods demonstrated higher detection efficacy for moor macaques compared to point transects, their implementation requires careful consideration given their logistical and financial constraints. The primary limitation is the substantial initial investment in equipment, which may hinder monitoring projects with limited budgets (Speaker et al. 2022; Table 1), although the cost of equipment is constantly declining. However, this initial cost can be outweighed over long‐term studies, as the reduced field effort compared to human‐based point transects could lead to significant savings in personnel‐related expenses (Kays et al. 2020). A second major consideration is data management. Camera traps and PAM generate large volumes of data, necessitating considerable post‐processing effort to identify and detect target species (Tuia et al. 2022). Manual processing of large‐scale monitoring projects may require weeks of work (Table 1). This bottleneck, however, is being alleviated by increasingly efficient and freely accessible deep‐learning models for automated detection of both camera trap images and vocalizations in audio recording, which can reduce processing time to under a day in some cases (Cowans et al. 2024; Darras et al. 2024). We recommend that monitoring programs conduct a thorough assessment of these economic and logistical factors during the planning phase to ensure project feasibility and long‐term success. Furthermore, both global and regional funding structures are needed to support the initial acquisition of new monitoring technologies, thereby enhancing the capacity for effective wildlife monitoring (White et al. 2022).

The effect of sampling method on moor macaque detectability highlights the importance of conducting pilot studies prior to surveys, as detectability affects the reliability of population estimates (MacKenzie et al. 2017; Ross and Reeve 2011). Based on our findings, we recommend using point transects as an initial approach to confirm primate species presence with minimal survey effort. If the species remains undetected or requires extensive effort, camera traps or passive acoustic monitoring (PAM) should be employed to increase survey coverage and detection probability. For rare and endangered species with low detectability, we recommend combining camera traps and PAM to maximize detection success. Discriminating between sampling methods before undertaking surveys may also contribute to reducing field effort and costs (Hermoso et al. 2013), which often critically affect the viability and logistics of population surveys (de Figueiredo Machado et al. 2023). Therefore, our study contributes to the design of more effective protocols to monitor targeted primate species, which are necessary to plan effective conservation strategies for threatened species (Jambari et al. 2023).

## Supporting information


**S1.** Frequency of moor macaque detection for each survey method and habitat type (Point transects (A–B): N_Forest_ = 12, N_Open_ = 11; Camera traps (C–D): N_Forest_ = 171, N_Open_ = 97; PAM (E‐F): N_Forest_ = 87, N_Open_ = 56) in both study areas (Hutan Pendidikan and TAHURA Bontobahari) as a function of the hour of the day. Point transect collected data from 6:00 to 18:00, and camera trap and PAM 24 h/day. Grey areas correspond to the night hours and yellow areas to the light hours. **S2.** Estimated moor macaque detection probability from the most parsimonious detection model, with standard errors (red bars) for the three survey methods tested (point transects: PT, *N* = 7; passive acoustic monitoring: PAM, *N* = 7; and camera traps: CT, *N* = 7) restricted to the 7 sampling locations where we collected data with PAM. Dash line represents moor macaque detection probability of 50%.
