# Supplementary material for: Continuous Audio‐Visual Sensor Monitoring Is More Effective Than Human Observers for Detecting Moor Macaques
Source: Am J Primatol. 2026 Jan 20;88(1):e70121. doi: 10.1002/ajp.70121 (PMC12888071; doi:10.1002/ajp.70121)

**Supplementary material**

**S1.** Frequency of moor macaque detection for each survey method and habitat type (Point transects (A-B): N_Forest_ = 12, N_Open_ = 11; Camera traps (C-D): N_Forest_ = 171, N_Open_ = 97; PAM (E-F): N_Forest_ = 87, N_Open_ = 56) in both study areas (Hutan Pendidikan and TAHURA Bontobahari) as a function of the hour of the day. Point transect collected data from 6:00 to 18:00, and camera trap and PAM 24h/day. Grey areas correspond to the night hours and yellow areas to the light hours.


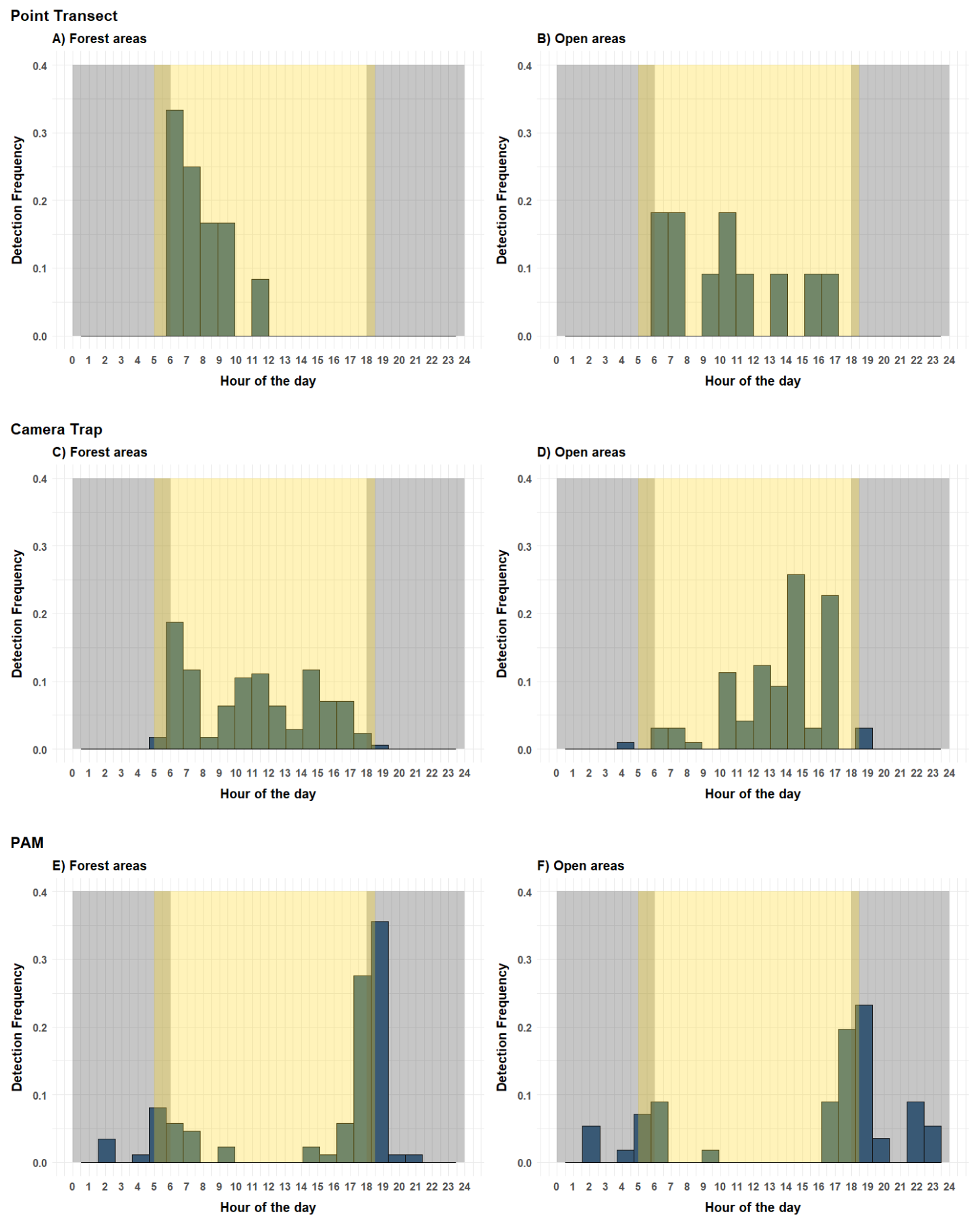


**S2.** Estimated moor macaque detection probability from the most parsimonious detection model, with standard errors (red bars) for the three survey methods tested (point transects: PT, N = 7; passive acoustic monitoring: PAM, N = 7; and camera traps: CT, N = 7) restricted to the 7 sampling locations where we collected data with PAM. Dash line represents moor macaque detection probability of 50%.


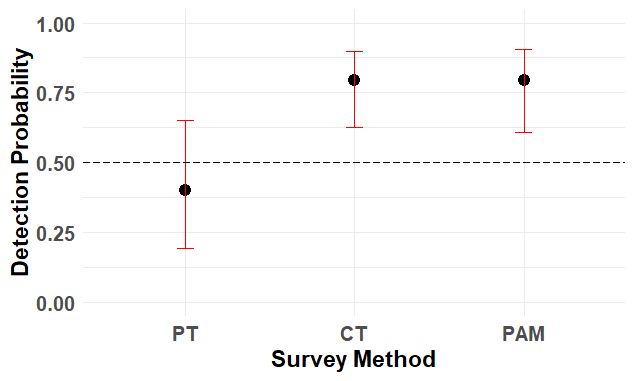

Supplement: Supplementary file 1 — S1. Frequency of moor macaque detection for each survey method and habitat type (Point transects (A–B): NForest = 12, NOpen = 11; Camera traps (C–D): NForest = 171, NOpen = 97; PAM (E‐F): NForest = 87, NOpen = 56) in both study areas (Hutan Pendidikan and TAHURA Bontobahari) as a function of the hour of the day. Point transect collected data from 6:00 to 18:00, and camera trap and PAM 24 h/day. Grey areas correspond to the night hours and yellow areas to the light hours. S2. Estimated moor macaque detection probability from the most parsimonious detection model, with standard errors (red bars) for the three survey methods tested (point transects: PT, N = 7; passive acoustic monitoring: PAM, N = 7; and camera traps: CT, N = 7) restricted to the 7 sampling locations where we collected data with PAM. Dash line represents moor macaque detection probability of 50%. [file AJP-88-e70121-s001.docx]
